# The Relevance of MicroRNAs in the Pathogenesis and Prognosis of HCV-Disease: The Emergent Role of miR-17-92 in Cryoglobulinemic Vasculitis

**DOI:** 10.3390/v12121364

**Published:** 2020-11-29

**Authors:** Serena Lorini, Laura Gragnani, Anna Linda Zignego

**Affiliations:** MASVE Interdepartmental Hepatology Center, Department of Experimental and Clinical Medicine, University of Florence, Center for Research and Innovation CRIA-MASVE, 50134 Firenze, Italy; serena.lorini@unifi.it (S.L.); laura.gragnani@unifi.it (L.G.)

**Keywords:** HCV, MIRNAs, HCC, NHL, cryoglobulinemic vasculitis, miR 17-92

## Abstract

Hepatitis C virus (HCV) is a major public health problem. HCV is a hepatotropic and lymphotropic virus that leads to hepatocellular carcinoma (HCC) and lymphoproliferative disorders such as cryoglobulinemic vasculitis (CV) and non-Hodgkin’s lymphoma (NHL). The molecular mechanisms by which HCV induces these diseases are not fully understood. MicroRNAs (miRNAs) are small non-coding molecules that negatively regulate post-transcriptional gene expression by decreasing their target gene expression. We will attempt to summarize the current knowledge on the role of miRNAs in the HCV life cycle, HCV-related HCC, and lymphoproliferative disorders, focusing on both the functional effects of their deregulation as well as on their putative role as biomarkers, based on association analyses. We will also provide original new data regarding the miR 17-92 cluster in chronically infected HCV patients with and without lymphoproliferative disorders who underwent antiviral therapy. All of the cluster members were significantly upregulated in CV patients compared to patients without CV and significantly decreased in those who achieved vasculitis clinical remission after viral eradication. To conclude, miRNAs play an important role in HCV infection and related oncogenic processes, but their molecular pathways are not completely clear. In some cases, they may be potential therapeutic targets or non-invasive biomarkers of tumor progression.

## 1. Introduction

Hepatitis C virus (HCV) is a major public health problem with 71 million infected people worldwide [1]. HCV is a hepatotropic and lymphotropic virus that can establish chronic infection, leading to hepatocellular carcinoma (HCC) and lymphoproliferative disorders (LPDs) such as mixed cryoglobulinemia (MC) and non-Hodgkin’s lymphoma (NHL). Only 19% of people infected with HCV are aware of their hepatitis status. The World Health Organization estimates that approximately 399,000 people died from HCV in 2016 (https://www.who.int/news-room/fact-sheets).

MicroRNAs (miRNAs) are small non-coding molecules made up of 21–23 nucleotides that negatively regulate post-transcriptional gene expression by decreasing their target genes expression [2]. MiRNAs can be isolated from cells and tissues and from bodily fluids such as serum, plasma, or urine [3], and are involved in many biological functions such as embryogenesis, organogenesis, metabolism, and apoptosis [3]. A possible role of miRNAs in intercellular communication has also been proposed [4] as they can enter the cells through the gap junction, either by passive or active transport [5]. Alterations in miRNA expression patterns have been reported in human pathologies including cancer, cardiovascular and metabolic diseases, diabetes, and viral infection [6].

In this review, we will summarize some aspects of miRNAs to HCV-induced diseases, focusing on both the functional effects of their deregulation as well as on their putative role as biomarkers based on association analyses. We will also provide original new data regarding the miR 17-92 cluster in chronically infected HCV patients with and without lymphoproliferative disorders.

## 2. MicroRNAs in the HCV Life Cycle

The most studied liver-specific miR-122 represents 70% of the total miRNA in liver-tissue [7,8]. MiR-122 supports HCV replication through the binding of two different sites in viral mRNA, which are located in the 5′-noncoding region [9]. In fact, these two binding sites called S1 and S2 [9] are close to the Internal Ribosome Entry Sites (IRES) sequences, and it was speculated that miR-122 could increase translation and polyprotein production [10]. 

Furthermore, it was found that miR-122 works together with the Argonaute protein (AGO), by stabilizing and protecting the uncapped HCV RNA from degradation [11]. 

Thus, the activation of translation via IRES is promoted by the AGO protein containing the miRNA-induced silencing complex (miRISC). The interaction between miR-122-miRISC and HCV-RNA results in miR-122 sequestration, preventing its binding with host targets and promoting HCV replication [12]. 

This knowledge led to the development of a miR-122 inhibitor, named miravirsen, as an experimental anti-HCV drug. Although miravirsen demonstrated long-term safety in 27 HCV patients enrolled in a phase II study, [13,14] the high efficacy and the absence of side effects resulting from direct acting antivirals (DAAs) halted research on this promising therapeutic agent.

An in vitro study showed the interaction between miR-122 and a host protein, DDB1-CUL4 associated factor 1 (DCAF1), which is involved in DNA replication, cell cycle regulation, proliferation, and DNA damage responses [15]. The authors investigated the role of DCAF1 in hepatocyte cell lines infected with a HCV replicon and observed that DCAF1 negatively regulates HCV IRES-mediated translation. Thus, miR-122 was downregulated by DCAF1 knockdown and when overexpressed, it restored HCV replication, intimating that DCAF1 is involved in HCV replication through the regulation of miR-122 [15]. Further in vivo analyses are necessary in order to confirm these in vitro results and to ascertain their physiological significance.

Moreover, a variant of HCV genotype 2 carrying a G28A substitution in the 5′ end exhibits efficient RNA replication in the absence of miR-122, while this mutation does not occur when miR-122 levels are abundant [16]. In fact, Ono and colleagues demonstrated that in the absence of miR-122, other miRNAs could interact with the HCV genome promoting its replication and this may be relevant in the pathogenesis of extrahepatic manifestations [12].

A wide range of microRNAs seem to be involved in the HCV cell cycle, acting as replication suppressors or stimulators. Several in vitro studies [17,18] showed that microRNAs could support HCV replication through the suppression of NF-kB signaling or by stimulating pro-survival pathways such as PI3K/Akt, Ras/ERK, and Wnt/-catenin signaling. Ishida and colleagues reported that, in hepatoma cell lines, the maintenance of low levels of miR-491 was capable of enhancing the replication of the HCV replicon as well as HCV itself [19]. When miR-491 was artificially restored, it inhibited the PI3 kinase/Akt pathway, showing a HCV-mediated mechanism of liver injury progression [19]. 

MiR-130a is significantly downregulated in HCV infected cell lines, and in vitro inhibits HCV replication [20]. The artificially induced over-expression of miR-130a upregulates the expression of type I IFN and other molecules involved in innate immune response and decreases the expression of miR-122, which is a well-defined miRNA boosting HCV replication [20]. 

In a recent study, the authors identified an actor of HCV maintenance in the autophagy process [21]. Amongst the genes involved in the autophagy process, ATG5, a key regulator of the process, was a target of miR-130a. ATG5 upregulates HCV replication through the downregulation of interferon stimulated gene expression [22]. Therefore, the downregulation of miR-130a induced by HCV appears to be another mechanism that indirectly supports viral replication repressing innate immunity. Moreover, an in vitro study revealed that miR-130a seemed to target an isoform of pyruvate kinase specific to the liver and red blood cells (PKLR) [23]. The authors speculated that by decreasing PKLR and pyruvate levels, as well as subsequent glycolysis levels, HCV replication is inhibited through the reduced production of ATP and other glycolytic intermediates. This was confirmed by the artificial PKLR downregulation that affected the production of pyruvate, negatively impacting HCV replication [23]. 

Another miRNA, miR-125b-5p, is able to control HCV replication through one of its targets, the human antigen R (HuR), which is an important host factor [24]. Indeed, miR-125b-5p was found to be upregulated in the serum of HCV infected patients and in replicon infected HCC cell lines. In vitro overexpression of miR-125b-5p decreases HCV RNA levels by up to 70% compared to controls. Conversely, Dai et al. reported that the inhibition of miR-125b-5p decreased HCV expression at both RNA and protein levels [25]. The authors showed that overexpression of miR-125b-5p is probably induced by interleukine6 (IL6)-signal transducer and the activator of transcription 3 (STAT3) signaling [25], while STAT3 is activated by the HCV core protein during HCV infection [26]. In light of these results, further studies are needed to elucidate and define the clinical role of miR-125b-5p during HCV infection.

The microRNAs involved in the HCV life cycle and their biological significance are reported in Table 1.

## 3. MicroRNAs in HCV Related HCC

According to GLOBOCAN 2018, liver cancer causes about 781,631 deaths, about 8.2% of the total number of cancer deaths and approximately 841,080 new liver cancers were diagnosed worldwide in 2018 [27]. HCV is responsible for 140,000 new cases of hepatocellular carcinoma (HCC), which is the primary liver cancer [28]. The importance of an early diagnosis has been demonstrated to improve the survival of HCC patients [29,30,31,32] even though, at present, the HCC guidelines report imaging-based diagnosis as the only option [33,34]. 

DAAs are the current standard of care in HCV treatment and have caused a reduction in HCC incidence [35], although a residual risk still remains in cases with advanced fibrosis and cirrhosis [36].

In this context, much effort has been made to find a predictive/early detection non-invasive biomarker, and miRNAs could be one of the potential candidates. 

Several studies tried to identify a panel of microRNAs to discriminate HCV-related HCC from cirrhosis with encouraging results [37,38,39]. 

Thus, the detection of predictive biomarkers of HCC development is an urgent issue. In a recent study comparing HCV patients with HCC to HCV patients with liver cirrhosis (LC), it was found that four miRNAs (miR-122-5p, miR-331-3p, miR-494-3p, miR-224-5p) significantly increased and two (miR-185-5p, miR-23b-3p) significantly decreased in HCC patients compared to LC patients [40]. The six miRNAs could efficiently discriminate HCC from LC, chronic hepatitis, and healthy controls. In addition to this recent analysis, other studies evaluated different miRNA combinations in order to discriminate HCV patients with cirrhosis from those who are at risk of developing HCC [41,42,43,44,45]. MiR-301 seems to be a promising molecule with 78.57% sensitivity and 89.58% specificity in distinguishing HCV-HCC from HCV chronic hepatitis [46]. MiR-301 levels were significantly linked with tumor size and serum alpha-fetoprotein (AFP) [46]. In a similar way, a combination of miR-224 with AFP shows sensitivity, specificity, and accuracy of 95.0%, 92.1%, and 93.2%, respectively in discriminating HCC from LC [47].

A microarray analysis of 2555 miRNAs in pre- and post- treatment HCC serum samples from 12 HCV patients, showed fluctuating levels of miR-125a-5p depending on the presence of HCC, therefore, presenting this molecule as a non-invasive biomarker for the diagnosis of early-stage cancer [48]. 

Another microarray analysis, performed on the serum samples of HCV patients, identified miR-3197 as a potential biomarker for cancer surveillance in patients who developed HCC after DAA therapy [49]. An expression study performed by Tamori et al. in HCC tissue samples revealed a different pattern between HCV positive patients (HCV-HCC) and those who successfully achieved sustained virological response (SVR-HCC) [50]. In fact, the authors showed the expression of some miRNAs that were classified into two opposite categories: oncomiR and anti-oncomiR. The oncomiR group includes microRNAs that play a role in tumor development and the anti-oncomir group includes molecules that negatively regulate cancer pathways. The difference in specific microRNA expression patterns between HCV-HCC and SVR-HCC samples confirms the role of previously identified oncomiR or anti-oncomiR in other malignancies, suggesting several cancer-related pathways [50]. 

A particular role seems to be played in HCC carcinogenesis by miR-138, a tumor suppressor with pleiotropic roles [51]. 

An in vitro study proposed a viral directly-induced carcinogenesis through the downregulation of tumor-suppressive miR-138 mediated by the HCV core protein [52]. In fact, miR-138 targets the telomerase reverse transcriptase, inhibiting its activity and inducing cell senescence, as planned in the physiological cell fate. The downregulation of miR-138 prevents the senescence process prolonging cell life [51]. In HCC Huh7 cell lines, the presence of a mature HCV core protein increases telomerase activity protecting the cells from miR-138 induced senescence. An in vitro study by Wang and colleagues showed miR-138 acts as a tumor suppressor, lowering the expression of human cyclin D3 and promoting cell cycle arrest. As the dual-luciferase reporter gene assay results showed that cyclin D3 was a direct target of miR-138 (a potential), a selective use of mir-138 mimic for the treatment of HCV-associated HCC is conceivable [53]. 

The HCV core protein seems to be involved in carcinogenesis through different pathways. In an in vitro study performed by Xu H et al., the overexpression of miR-196a in HCC cell lines promoted G1/S transition and subsequently cell proliferation [54]. The authors, in line with a previous study on cervical cancer [55], confirmed that forkhead box O transcription factor 1 (FOXO1) serves as a target of miR-196a. FOXO1 is a powerful transcription factor that plays an important role in various cellular functions including proliferation, differentiation, cell survival, longevity, and oxidative stress resistance [56]. When miR-196a is downregulated, HCC cell proliferation is increased [54]. 

Many different microRNAs have been proposed as non-invasive biomarkers of liver damage and tumor progression [57]. 

Piluso et al. found a significant alteration of miR-21 and miR-26b in HCV-related HCC patients when compared to healthy subjects, chronic HCV, and HCV-NHL patients, suggesting their use as non-invasive biomarkers and their involvement in HCV-related hepatic malignancies [58].

Rashad and collaborators showed that miR-18a and miR-27a expression levels in blood samples could aid in the discrimination between HCV-related cirrhosis and HCC [59]. MiR-27 was found to be downregulated in the carcinoma tissue of HCV infected patients together with miR-199, miR-200, and let-7 [60]. A human cancer-related miR-135, which is already known to be involved in HCV replication [61], targets the tumor suppressor protein tyrosine phosphatase receptor delta (PTPRD). PTPRD was inversely correlated with the STAT3 protein which was found activated in 60% of HCC patients [62], suggesting a pivotal role in liver cancer progression.

Another aspect in microRNAs research is the presence of miRNAs’ allelic variants due to single nucleotide polymorphisms (SNPs) that could tune and change their effects on targets. The available literature regarding the effects of SNPs in microRNA coding genes in viral-related HCC is scarce and reports controversial results.

The G allele frequency of miR-499 A > G SNP (rs3746444) was significantly lower in HCC Egyptian patients [63], whereas, on the contrary, another study showed no correlation between miR-499 genotypes and HCC in the Caucasian, Asian, and Chinese populations [64]. Another controlled analysis on Egyptian HCC patients, conducted in a small number of cases (thirty-seven HCC, forty-six HCV-chronically infected subjects, and thirty-two healthy controls) associated miR-101-1 CC genotype with an increased risk of liver cancer development in patients [65]. 

This attempts to outline a complex situation that requires further analyses on wider populations that take into account the different distributions of allelic variants in various ethnic groups and that will correct the results based on this variability. 

MicroRNAs involved in HCV-related HCC and their putative pathways are detailed in Table 2.

## 4. MicroRNAs in HCV-Related Lymphomas and Lymphoproliferative Disorders

HCV infection has a strong causal association with LPDs and amongst them, the most frequent is an autommune/B-cell LPD called mixed cryoglobulinemia (MC) [66]. MC is the most described and well documented HCV-related extrahepatic disorder [67], and is a benign condition that can evolve into malignancy in 8–10% of cases [67,68]. Moreover, MC patients had a 35 times higher risk of developing NHL than the general population [69]. MC is characterized by the presence of circulating immunoglobulins that become insoluble below 37 °C (cryoglobulins-CGs), which cause a systemic vasculitis called cryoglobulinemic vasculitis (CV) in small/medium-sized vessels, therefore, affecting many organs and tissues [67]. The majority of CV patients obtain vasculitis remission after a SVR due to antiviral treatment, although, in some cases, symptoms persist or recur after a SVR [70,71,72,73].

At present, there are few studies regarding the involvement of miRNAs in HCV-related LPDs [74]. The first analysis on this topic used a microarray approach to evaluate the expression profile of 381 miRNAs in 15 tissue samples of splenic marginal zone lymphomas and from 11 non-neoplastic splenic tissue samples [75]. Five miRNAs (miR-21, miR-155, miR-146a, miR-494, and miR-34a) were found significantly overexpressed, whereas seven miRNAs (miR-139, miR-345, miR-125a, miR-126, miR-26b, miR-138, and miR-95) showed a significantly reduced expression in the lymphoma tissue. In particular, miR-26b, a miRNA known to have tumor suppressive properties, was significantly downregulated in lymphomas arising in HCV-positive patients [75]. A subsequent study from Fognani E. et al. analyzed the expression profile of six miRNAs (miR-Let-7d, miR-16, miR-21, miR-26b, miR-146a, and miR-155) in peripheral blood mononuclear cells (PBMCs) of 167 HCV patients and 35 healthy controls [76]. The authors observed the downregulation of miR-26b in two subgroups of patients: HCV-CV and HCV-NHL patients and the restoration of miR-26b levels in a subgroup of CV patients after a treatment-induced clinical remission of vasculitis [76]. Moreover, Piluso et al. confirmed the alteration of miR-16, miR-21, miR-26b, and miR-155 in the PBMCs of HCV-NHL patients [58]. Whereas, miR-26b was downregulated, the other three miRNAs were upregulated when compared to healthy subjects and HCV patients without malignancies [58]. These results appear rather interesting in light of a substantially recent finding that attributed a tumor suppressing ability to miR-26b in different kinds of cancers [77,78,79]. The putative targets and functional effectors of miR-26b are the lymphoid enhancer binding factor 1 (LEF1) [80], overexpressed in chronic lymphocytic leukemia and in the monoclonal B cells lymphocytosis, a pre-lymphomatous condition [81], and cyclooxygenase-2, COX2 [79]. In this scenario, the downregulation of miR-26b associated with CV and NHL in HCV-positive patients, could be an indicator of a molecular mechanism of tumor progression [82]. 

In addition, Fognani and co-workers described a significant increase in the expression of three oncomiRs, miR-21, miR-155, and miR-16 in PBMCs from the HCV-NHL subgroup. MiR-21 and miR-155 were previously associated with pro-survival activity and autoimmunity disorders [83,84] and recent evidence has suggested that miR-155 could be a therapeutic target in patients with both HCV-chronic infection and leukemia [85]. In fact, the authors performed an in vitro knockout of miR-155 in primary lymphoid cultures from pediatric patients and observed an inhibition on leukemic cell proliferation and viral replication [85]. MiR-155 expression is controlled by a wide range of signaling pathways, but its role as oncomiR is well defined in immune response and inflammation [86]. The aberrant transformation of lymphoid cells could be induced by immune system chronic stimulation due to HCV persistence, leading to an upregulation of miR-155 expression. Further studies are required to elucidate the promising therapeutic use of miR-155 inhibitor.

Another analysis performed on lymphoma tissue samples, revealed 52 miRNAs differentially expressed in HCV-associated diffuse large B cell lymphoma (DLBCL) [87]. Amongst the 52 microRNAs, the authors identified four of them (miR-138, miR-147a, miR-147b, and miR-511) as predictors of the overall survival in HCV-associated DLBCL, highlighting their potential as prognostic or therapeutics biomarkers [87].

Conversely, Bruni et al., who analyzed 34 miRNAs in formalin-fixed paraffin-embedded tissues from HBV/HCV positive and negative patients with indolent NHL [88], did not confirm the previously reported miRNA dysregulations. In fact, the authors showed miR-92a downregulation in HBV/HCV negative samples and miR-30b overexpression only in HCV positive tissue, attributing the discrepancy with previously published results to the limited number of cases [88]. 

MicroRNAs involved in HCV-related LPDs and their deregulations are summarized in Table 3.

## 5. Cluster miR 17-92 in HCV-Related Cryoglobulinemic Vasculitis

MiR 17-92 is a cluster of microRNAs transcribed from a polycistronic gene, comprising of 6 different mature microRNAs: miR-17, miR-18, miR-19a, miR-19b, miR-20a, and miR-92a [89] that was found overexpressed in lymphoma cell lines, and in patients with DLBCL for the first time [89,90]. MiR 17-92, which is also known as oncomiR-1, was the first microRNA to which an oncogenic role was attributed [89]. O’Donnell and collaborators showed that the transcription of miR 17-92 is activated directly by the oncogene c-Myc [91] and, in fact, in transgenic c-myc overexpressing mice, an increase in miR 17-92 expression levels can be observed in parallel [92]. Overall, an upregulation has been observed in a wide variety of lymphatic neoplasms [93,94], but also, in various solid tumors, such as gastric [95], lung [96], thyroid [97], and HCC [98,99]. In addition to neoplastic diseases, the overexpression of miR 17-92 has been observed in LPDs and its role has also been hypothesized in the regulation of B cell [100] and T cell functions [101].

To date, scarce information is available regarding the cluster miRNA 17-92 and HCV-related disorders and only Shrivastava et al. reported that miR-20a and miR-92a expression levels correlated with fibrosis stage in HCV-infected patients and decreased in resolved infections, suggesting their use as predictive biomarkers [102].

The preliminary results concerning the expression profile of the miR 17-92 cluster in HCV positive patients with and without CV are reported here. The aim of this retrospective study was to evaluate the role of the cluster as a biomarker in HCV-related LPDs.

### 5.1. Patients and Methods

At the Department of Internal Medicine, Center for Systemic Manifestations of Hepatitis Viruses (MaSVE) at the University of Florence, 79 chronically infected HCV patients were recruited: 34 HCV without signs/symptoms of LPDs and/or autoimmune disease (HCV), 45 HCV with signs/symptoms of CV. All the patients successfully underwent DAAs-based antiviral therapy and obtained a SVR. For 20/45 CV subjects, PBMCs samples were available before treatment and 6 months after the end of therapy. The main demographic, clinical, and laboratory data are reported in Table 4. Female sex was prevalent in the CV group, as it was expected, since the autoimmune rheumatic diseases are more frequent in women [103,104]. HCV infection was proven by detecting HCV RNA (AMPLICOR HCV Test, v2.0; Roche Diagnostics, Alameda, CA- USA). The HCV genotype was assessed by the VERSANT HCV Genotype 2.0 assay (Siemens Healthcare Diagnostics, Deerfield, IL- USA). CV was assessed as previously described [105]. The study was conducted in accordance with the ethical guidelines according to the Declaration of Helsinki.

PBMCs were isolated from fresh anticoagulated blood by gradient precipitation on Lymphoprep (Axis-Shield PoC AS, Oslo, Norway) according to the manufacturer’s instructions. After the second wash, the cells were counted and stored at −80 °C. Total RNA was extracted from 5 × 106 PBMCs using Trizol reagent (Invitrogen, USA) according to the manufacturer’s instructions. C. elegans miR-39 synthetic RNA oligonucleotide (1.1 × 108 copies/106 cells) was added to PBMCs samples and used as an external control to monitor extraction efficiency.

In order to evaluate the expression levels of each member of the cluster, we performed a primer specific retrotranscription using the TaqMan MicroRNA Kit (Applied Biosystems, CA, USA) and 160 ng of total RNA. Through real-time PCR, we evaluated the expression levels of miR-Let-7d, miR-17, miR-18a, miR-19a, miR-19b, miR-20a, and miR-92a using specific TaqMan MicroRNA Assays (Applied Biosystems, CA, USA). Relative expression levels of the different miRNAs were evaluated by using the 2-ΔΔct method [106] and MiR-Let-7d was used as an endogenous control to normalize the results as previously described [76]. 

C-Myc expression levels were assessed by real-time PCR using a commercial assay (Applied Biosystems, CA, USA) and the results were analyzed using the 2^-ΔΔct^ method [106] with β-actin and GAPDH as housekeeping genes. 

Data are expressed as mean ± SD. The quantitative variables were analyzed using analysis of variance (non-parametric ANOVA). All tests were 2-sided, and *p* values less than 0.05 were considered significant. Analyses were performed using Stata v.9.0 (StataCorpLP, College Station, TX, USA).

### 5.2. Results

**Figure 1:**
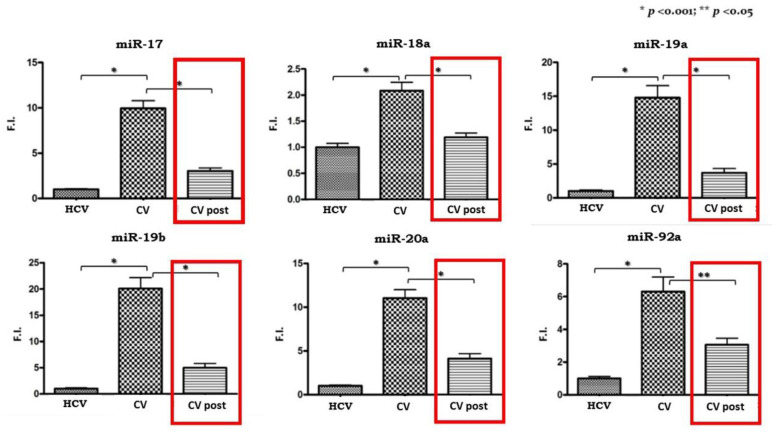
Expression levels of miR 17-92 cluster in pre-therapy Peripheral Blood Mononuclear Cells (PBMCs) samples from the HCV group and in the pre-Cryoglobulinemic Vasculitis (CV) and post-therapy (CV post) PBMCs samples from the CV group. On the Y axis miRNA expression levels are reported as fold increase changes. * *p*-value < 0.001; ** *p*-value < 0.05.

All of the cluster members were significantly upregulated in PBMCs isolated from CV patients compared to the HCV control subjects as shown in Figure 1.

Subsequently, we analyzed the expression levels of miR 17-92 in the 20 CV patients who obtained a SVR after antiviral therapy. We compared pre- and post-therapy PBMCs samples and the results are shown in Figure 1. A significant reduction in expression levels can be observed for all the cluster members, although they do not reach levels comparable to those of pre-therapy in the control group.

With the aim of confirming the link between cluster upregulation and c-Myc expression, we evaluated its mRNA levels in PBMCs samples from the same patients. C-Myc expression levels were doubled in CV patients compared to the HCV control group, and just like the miRNA 17-92 cluster, they decreased in the CV post-therapy samples.

## 6. Discussion

In this paper, we have reported some of the several findings concerning the involvement of microRNAs in different aspects of HCV infection, from the contribution of these host epigenetic regulators in the viral life cycle to the effects that their deregulation, directly or indirectly induced by HCV, could have on the pathogenesis of HCC and LPDs.

The discovery of a causal mechanism induced by a specific miRNA opens up new therapeutic approaches and, although this is no longer a priority regarding HCV-therapy as powerful and safe antiviral drugs are now available, effective drugs for HCV-induced HCC and lymphomas are still lacking. Likewise, tools to make an early diagnosis and to accurately formulate a prognosis could be extremely helpful in the clinical management of patients with virus-related cancers. 

For these reasons, researchers are still focused on the study of microRNAs, although, as is evident in the literature and in our review, the results are often confounding or controversial and are, therefore, hard to interpret. 

The original unpublished results reported here showed, for the first time, that the expression pattern of all the members of the miRNA cluster 17/92 is significantly increased in PBMCs isolated from patients with HCV-related CV. This is in line with previous results that attributed a functional role of cluster members in LPD onset [90,94], with a special focus on B-lymphoproliferative/autoimmune disorders [100]. The significant reduction in the expression levels of miRNAs following viral eradication, reinforced the speculation that the 17/92 cluster plays an important role in the pathogenesis of HCV-related CV.

In addition, we found elevated baseline levels of c-Myc mRNA in CV patients compared to those with HCV infection without LPDs. Although the results were close, they did not reach statistical significance, perhaps due to the number of samples. This upregulation seems to confirm the role of c-Myc in activating the transcription of the various members of the cluster, as its mRNA levels decreased after a therapy-induced virological and clinical response. In fact, the detection of c-Myc rearrangement causing protein overexpression in cryoglobulinemic patients was reported as an early event in malignant B cell transformation [107]. In this light, the transcription of miR 17-92 could be considered as a downstream event consequent to the rearrangement and overexpression of c-Myc.

Overall, especially in the model of HCV infection, the microRNA field of research deserves to be continued and improved upon, in order to reach solid and reproducible findings that are able to define new pathogenetic hypotheses, in addition to establishing prognostic and therapeutic strategies that could be useful in different conditions.

## Figures and Tables

**Table 1:** MicroRNAs involved in the Hepatitis C Virus (HCV) life cycle and their pathways.

Name	Inhibition/Enhancement of HCV Replication	Pathways	Ref
miR-122	enhancement	Binds to S1 and S2 sites at the 5′ non coding region on viral RNA and increases translation	[9,10]
		Together with the Argonaute protein (AGO) stabilizes and protects the uncapped viral genome from degradation	[11]
		Interaction between miR-122-miRISC-HCV-RNA results in miR-122 sequestration, preventing its binding with host targets and promoting HCV replication	[12]
		DCAF1 targets miR-122 and negatively regulates HCV IRES-mediated translation	[15]
miR-125b-5p	Controversial	Downregulates HuR which promotes HCV replication	[24]
		Inhibits miR-125b-5p decreasing HCV expression at both RNA and protein levels	[25]
miR-130a	inhibition	Inhibits the ATG5 protein that upregulates the expression of type I IFN and of molecules involved in innate immune response	[20,21,22]
		HCV replication is inhibited through the reduced production of ATP and other glycolytic intermediates	[23]
miR-199a-5p	enhancement	Stimulates pro-survival pathways like PI3K/Akt, Ras/ERK, and Wnt/β-catenin	[18]
miR-215	enhancement	Inactivates NF-κB pathway by inhibiting TRIM22	[17]
miR-491	enhancement	Low levels of miR-491 reduced the inhibition of PI3K/AKT pathway which is involved in the maintenance of HCV replication	[19]

**Table 2:** MicroRNAs differentially regulated in HCV-related Hepatocellular Carcinoma (HCC.)

Name	Expression	Samples Type	Pathways/Putative Pathways	Ref.
miR-16	Down	serum samples	Suppresses invasion and migration of HCC cells through different targets	[39,45]
miR-17-5p	Up	Serum samples	Could suppress invasion and metastasis of oncogenic c-Myc in HCC cells	[42]
miR-18b	Up	serum samples	Unknown	[59]
miR-21	Up	PBMC samples	Increases tissue invasion targeting many tumor suppressors such as PTEN and PDCD4	[43,58]
miR-23b-3p	Down	serum samples	Acts as tumor suppressor, regulating migration and invasion by targeting Pyk2	[40]
miR-26b	Down	PBMC samples	Promotes apoptosis and tumor suppression, targeting different target genes	[58]
miR-27	Down	tissue samples	Targets genes involved in cell cycle and apoptosis	[60]
miR-27a	up	serum samples/ tissue samples	Leads to up-regulation of transcriptional factor specificity protein (Sp), vascular endothelial growth factor (VEGF), and VEGF receptor 1 (VEGFR1)	[59]
miR-29b	Down	serum samples	Suppresses tumor angiogenesis, invasion, and metastasis by regulating matrix metallo proteinase 2 expression	[38]
miR-30c-5p	Up	Serum samples	Suppresses cells migration and invasion in other types of solid tumors through different targets	[42]
miR-34a	Up	Serum samples	Inhibits cells proliferation targeting SATB2	[45]
miR-122-5p	Up	serum samples	Acts as tumor-suppressor in HCC development by targeting genes such as metalloprotease, BCL-w, and cyclin G1	[38,40]
miR-125a	Down	Serum samples	Inhibits HCC cell proliferation and induces apoptosis in vitro and in vivo	[45]
miR-125b	Down	serum samples	Suppresses HCC invasion/metastasis by targeting VEGF	[37]
miR-128	Up	tissue samples	Iinvolved in HCV life cycle	[60]
miR-135	Up	In vitro/ tissue samples	Inhibits the tumor suppressor protein tyrosine phosphatase receptor delta (PTPRD) and inversely correlates with STAT3 protein which was often activated in HCC	[62]
miR-138	Down	In vitro/serum samples/tissue samples	Inhibits the telomerase reverse transcriptase and induces cell senescence	[51,52]
miR-138b	Down	Serum samples	Unknown	[37]
miR-139	Down	serum samples	Inhibits proliferation and invasion of HCC cells	[45]
miR-143	Down	Serum samples	Negatively regulates proliferation and invasion targeting FGF1	[41]
miR-145	Down	serum samples	Is associated with inflammation fibrosis	[37,41,45]
miR-150	Down	serum samples	Negatively regulates proliferation and invasion targeting MMP4	[44]
miR-152	Down	serum samples	Targets the DNA methiltransferase-1 which sustains fibroblast activation	[57]
miR-155	Up	In vitro/tissue samples	Contributes to tumor progression targeting PTEN	[58]
miR-181a-c	Up	tissue samples	Promotes migration and invasion	[60]
miR-182	Down	serum samples	Promotes progression and angiogenesis	[44]
miR-185-5p	Down	serum samples	Acts as a tumor suppressor by targeting multiple genes	[40]
miR-196a	Up	In vitro	Facilitates cell proliferation by inducing the G1-S transition	[54]
miR-199	Down	tissue samples	Targets genes involved in cell cycle and apoptosis	[60]
miR-199a	Down	serum samples	Inhibits tumor progression through different pathways	[39,45]
miR-200	Down	tissue samples	Targets genes involved in cell cycle and apoptosis	[60]
miR-214-5p	Down	serum samples	Regulates fibroblast growth factor receptor 1 expression	[37]
miR-221	Up	Serum samples	Promotes tumor progression via PTEN/PI3K/AKT pathway	[45]
miR-223-3p	Down	Serum samples	Inhibits HCC cell proliferation and promotes apoptosis by directly targeting NLRP3	[42]
miR-224	Up	Serum samples	Putative oncogenic miRNA (not specified)	[47]
miR-224-5p	Up	serum samples	Putative oncogenic miRNA (not specified)	[40]
miR-301	Up	serum samples	Enhances stem cell traits in HCC cells and plays a crucial role in Tumor Associated Neutrophil-induced effects	[46]
miR-302c-3p	Up	Serum samples	Inhibits migration and invasion targeting TRAF4	[42]
miR-331-3p	Up	serum samples	Promotes proliferation and metastasis through the suppression of leucine-rich repeat protein phosphatase mediated dephosphorylation of AKT	[40]
miR-335-5p	Up	tissue samples	Is associated with non-alcoholic fatty liver disease and metabolic disorders	[60]
miR-375	Down	serum samples	Its downregulation mediated by β-catenin, promotes tumor formation through upregulation of YAP1, AEG-1, and of IGF1R, which induces cell growth	[37]
miR-494	Up	serum samples	Induces endothelial to mesenchymal transformation by targeting SIRT3/TGF-β/SMAD signaling	[37]
miR-494-3p	Up	serum samples	Promotes cell proliferation, migration, and invasion by targeting PTEN	[40]
miR-885-5p	Up	serum samples	Suppresses HCC by inhibiting Wnt/β catenin signaling	[38,43]
miR-1269	Up	serum samples	Promotes proliferations targeting FOXO1	[37]
miR-3197	Up	serum samples	Unknown	[49]
let-7-a1	Down	Serum samples	Targets genes involved in cell cycle and apoptosis	[41]
let-7 family	Down	tissue samples	Targets genes involved in cell cycle and apoptosis	[60]

**Table 3:** MicroRNAs differentially regulated in HCV-related lymphoproliferative disorders.

Name	Deregulation Type	Samples Type	Pathways/Putative Pathways	Ref
miR-16	Up	PBMC	Unknown	[58,76]
miR-21	Up	PBMC	Promotes NF-kappa B activity	[58,75,76]
miR-26b	Down	PBMC	May targets NEK6 that inhibits cellular senescence	[58,75,76,82]
miR-30a	Up	tissue samples	Suppresses proliferation and metastasis	[88]
miR-34a	Up	tissue samples	Downregulates FOXP1 during DNA damage response to limit BCR signaling	[75]
miR-92a	Down	tissue samples	Promotes tumor progression	[88]
miR-95	Down	tissue samples	Induces cell proliferation in other tumors through several targets	[75]
miR-125a	Down	tissue samples	Directly involved in NF-kB activity	[75]
miR-126	Down	tissue samples	Inhibits proliferation and apoptosis	[75]
miR-138	Down	tissue samples	Inhibits the telomerase reverse transcriptase and induces cell senescence	[75,87]
miR-139	Down	tissue samples	Involved in cell migration and proliferation	[75]
miR-146a	Up	tissue samples	Inhibits NF-kB driven inflammation	[75]
miR-147a	Up	tissue samples	Inhibits cell proliferations	[87]
miR-147b	Up	tissue samples	Stimulates proliferation and invasion	[87]
miR-155	Up	tissue samples/PBMC	Promotes NF-kappa B activity	[58,75,76]
miR-345	Down	tissue samples	Acts as a tumor suppressor in various malignancies	[75]
miR-494	Up	tissue samples	Promotes cell proliferation and invasion	[75]
miR-511	Up	tissue samples	Is a possible suppressor of cell proliferation	[87]

**Table 4:** Demographical, clinical, and serological characteristic of 79 HCV patients.

	HCV (*n* = 34)	CV (*n* = 45)
**Age** (years)	54.2 ± 11.9	62.5 ± 7.23
**Sex** (M/F)	26(76%)/ 8(24%)	11(24%)/ 34(76%)
**Histology**		
Chronic Hepatitis Cirrhosis	28 (82%) 6 (18%)	28 (62%) 17 (38%)
**ALT ^^^** (UI/L)	175 ± 126	161 ± 90
**HCV-RNA** (IU/mL × 10^6^)	3.6 ± 3.2	3.3 ± 2.8
**HCV genotype**		
1	22 (65%)	23 (51%)
2	6 (18%)	20 (44%)
3	5 (14%)	2 (5%)
4	1 (3%)	-
**Cryocrit**	0	11.7 ± 17.1
**C4 (mg/dL)**	19 ± 5	8 ± 7
**RF^†^ (IU/mL)**	-	273 ± 430

Results are reported as mean ± SD. ^ Alanine aminotransferase (ALT) normal values: 1265– IU/L; C4, normal value: 20–150 mg/dL; **^†^** Rheumatoid factor, normal value: < 20 IU/mL.
